# Nano-Engineered Biomimetic Optical Sensors for Glucose Monitoring in Diabetes

**DOI:** 10.3390/s16111931

**Published:** 2016-11-17

**Authors:** Sajid Rauf, Muhammad Azhar Hayat Nawaz, Mihaela Badea, Jean Louis Marty, Akhtar Hayat

**Affiliations:** 1 Interdisciplinary Research Centre in Biomedical Materials (IRCBM), COMSATS Institute of Information Technology, 54000 Lahore, Pakistan; sajidrauf@ciitlahore.edu.pk (S.R.); azharhayat@ciitlahore.edu.pk (M.A.H.N.); 2 Faculty of Medicine, Transilvania University of Brasov, 500019 Brasov, Romania; mihaela.badea@unitbv.ro; 3 BAE Laboratory, Universite de Perpignan Via Domitia, 66860 Perpignan, France

**Keywords:** nanotechnology, biomimetic nanomaterials, optical sensors, glucose monitoring, diabetes

## Abstract

Diabetes is a rapidly growing disease that can be monitored at an individual level by controlling the blood glucose level, hence minimizing the negative impact of the disease. Significant research efforts have been focused on the design of novel and improved technologies to overcome the limitations of existing glucose analysis methods. In this context, nanotechnology has enabled the diagnosis at the single cell and molecular level with the possibility of incorporation in advanced molecular diagnostic biochips. Recent years have witnessed the exploration and synthesis of various types of nanomaterials with enzyme-like properties, with their subsequent integration into the design of biomimetic optical sensors for glucose monitoring. This review paper will provide insights on the type, nature and synthesis of different biomimetic nanomaterials. Moreover, recent developments in the integration of these nanomaterials for optical glucose biosensing will be highlighted, with a final discussion on the challenges that must be addressed for successful implementation of these nano-devices in the clinical applications is presented.

## 1. Introduction

Diabetes mellitus (DM) is a chronic metabolic disorder that has emerged as a great socioeconomic burden for the developing countries. Currently, DM affects more than 240 million people around the world and this figure is expected to increase substantially to 380 million by 2025, with 80% of burden occurring in low- and middle-income countries. The important factors of DM diseases are a family history of DM, age, obesity, impaired glucose tolerance, gestational diabetes, and chronic hypo-glycaemia and hyper-glycaemia with disturbances in the metabolism of carbohydrates, fats and proteins resulting from defects in insulin secretion, insulin action or both [1,2]. DM can lead to complications such as coronary heart disease (CHD), retinopathy, nephropathy, stroke, kidney disease, blindness, dental disease and lower-limb amputations. This can also results into life threatening conditions including but are not limited to risks of cardiac, nervous, renal, ocular, cerebral and peripheral diseases [3,4]. Glucose plays an important function in the human body, where it serves as the primary energy source for the brain and is also as a source of energy for cells throughout the body. This energy helps the cells carry out nerve cell conduction, muscle cell contraction, active transport and the production of chemical substances [5]. 

Considering the burden, it adds to the frail health and economic systems of a developing country, there is a dire need to conduct research and develop comprehensive and cost-effective methodologies to address this disease. In this context, glucose sensors for continuous monitoring of glucose are considered a highly attractive area of scientific research to ensure public health safety [6]. The optical detection of glucose is mainly based on the conversion of glucose into gluconic acid and hydrogen peroxide in the presence of glucose oxidase. The peroxidase-catalyzed oxidation of the generated H_2_O_2_ in the presence of 3,3',5,5',-tetramethylbenzdine/2,2'-azino-bis(3-ethylbenzothiazoline-6-sulphonic acid) (TMB/ABTS) results in the formation of a colored product that can be monitored for colorimetric detection of glucose. Over the past decade, as an alternative to the natural enzyme peroxidase, a major fraction of the research has been devoted to the exploration of enzyme mimetics. With the advent of nanotechnology, various types of nanomaterials have been investigated in the literature towards construction of optical glucose sensors based on their oxidase- or peroxidase-like properties [7]. The mechanism of colorimetric detection of glucose using nanomaterial-based artificial enzymes is given below (see Equations (1) and (2)):

(1) $$\text{Glucose}+O_{2}\;\underset{\to }{\text{Glucose Oxidase}}\;H_{2}O_{2}+\text{Gluconic Acid}$$

(2) $$\text{TMB}+H_{2}O_{2}\;\underset{\to }{\text{Nanozymes}}\;\text{Oxidized TMB}+H_{2}O$$

The next section will focus on the advantages and disadvantages of enzymatic glucose sensors in order to help the reader compare the performance of non-enzymatic sensors with enzyme-based methodologies. 

## 2. Enzymatic Glucose Sensors: Advantages and Disadvantages

Generally, all the natural enzymes are proteins except for some catalytic RNA molecules, and are therefore prone to several intrinsic drawbacks. For example, they can undergo digestion by proteases, and they can degrade upon exposure to variable environmental conditions. Other disadvantages include time consuming preparation and purification processes, relatively high cost and the need for specific storage conditions. When considering enzymatic glucose sensors, there must be a balance between their advantages and disadvantages. Despite their huge industrial demand, enzymatic glucose sensors are not completely commercially viable and have a number of critical flaws. For example, first generation glucose sensors rely on the presence of oxygen, and are therefore hard to implement as reliable analytical tools for practical use. Moreover, they can be very easily exposed to interfering electroactive species. Alternatively, second generation glucose sensors based on the use of mediators were proposed to overcome the problem of oxygen dependency, and to offer lower amperometric potential to avoid the interference problems to some extent, but such sensor designs are elaborate, involve complicated fabrication methodologies and are unsuitable for mass production, limiting their commercial viability. In the same context, third generation glucose sensors are in the early phases of development, and there still is a lot to do to achieve the desirable analytical figures of merits [6]. However, despite of all these problems, enzymatic glucose sensors remain unchallengeable from a commercial point of view. 

Recently, nanomaterials mimicking traditional biological catalysts have attracted significant interest for their potential applications as artificial enzymes [7,8]. The high surface to volume ratio, high catalytic activity and abundance of reactive groups on their surface make these materials powerful candidates as alternatives to biological catalysts. Several types of engineered nanoparticles (NPs) have shown ‘enzyme-like’ activity, mostly as oxidase, peroxidase and catalase mimetics and some have been used as active materials in bioassays, biotechnology and in the biomedical field [9,10]. NP-based enzyme mimetics offer advantages in terms of cost, high stability, ease of production and tenability of catalytic activity. Keeping in view the important role of nanomaterials in (bio)sensor design, this review paper will focus on the analytical potential of biomimetic nanomaterials for the colorimetric detection of glucose in diabetes monitoring. We will discuss different types of nanomaterials employed in non-enzymatic assays according to their intrinsic nature and detection methodologies.

## 3. Types of Nanomaterials 

Over the past two decades, peroxidase-like nanomaterial-based artificial enzymes (nanozymes) coupled with glucose oxidase (GOx) have been frequently employed in the construction of glucose biosensors [7]. In such reactions peroxides, like hydrogen peroxidase and lipid peroxidase, are reduced and a redox substrate is oxidized by electron donation (Equation (3)):

(3) $$\begin{array}{l} 2\text{AH}+H_{2}O_{2}\;\underset{\to }{\text{Peroxidase}}\;2A+2H_{2}O\\ 2A+\text{ROOH}\;\underset{\to }{\text{Peroxidase}}\;2A+\text{ROH}+H_{2}O \end{array}$$

Horseradish peroxidase (HRP) is the most common example of the peroxidases enzyme family which is used as a peroxidase standard for peroxidation reactions due to its low substrate specificity [11]. The colorimetric detection of glucose, based on a redox reaction between HRP and colorimetric substrates such as TMB and ABTS has many advantages like high sensitivity, selectivity and simplicity. Colorimetric detection can be achieved even by the naked eye through the color changes of colorimetric substrates. Previously widely used electrochemical biosensors have drawbacks, especially in in vivo glucose sensing where endogenous electroactive species cause interferences. Sometimes cells surrounding the electrode are also damaged due to the use of electrochemical electrodes which results in a limited sensitivity [12,13].

**Figure 1:**
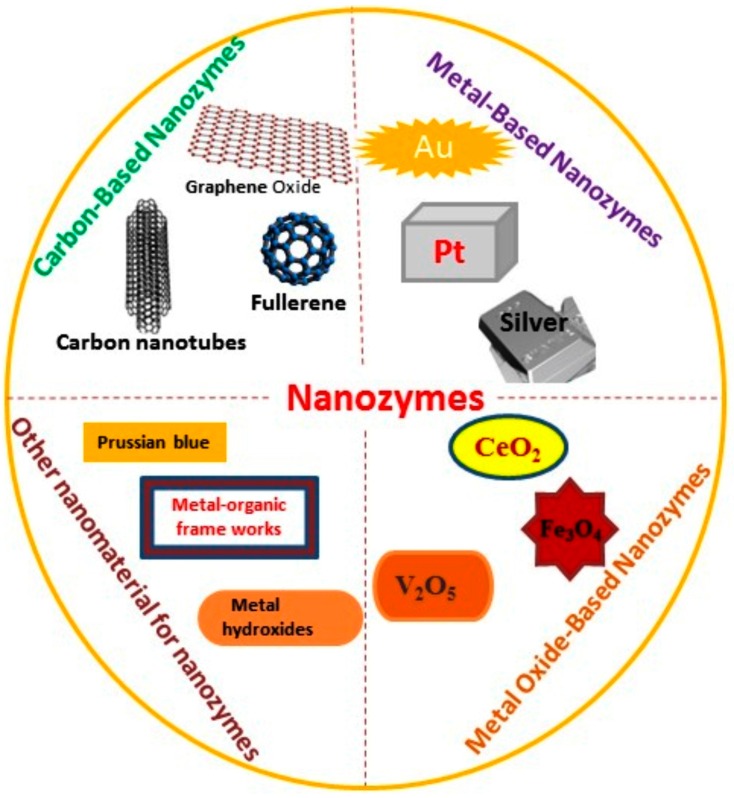
Classification of nanomaterial-based artificial enzymes (nanozymes).

A large number of nanozymes have been reported which mimic HRP for different applications. Herein, we focus on nanomaterials with peroxidase-like activity to develop optical detection system for glucose monitoring. Figure 1 provides a generic overview on different types of nanozymes that are potentially used to replace the natural enzyme in optical sensing methodologies for glucose monitoring.

### 3.1. Carbon-Based Nanozymes

Carbon-based nanomaterials such as graphene, carbon nanotubes, fullerene and their derivatives are fascinating nanomaterials that possess various applications in almost all domains of sciences [14]. Carbon-based nanomaterials have been extensively studied by researchers working in the field of nanozymes because of their exceptional catalytic properties. Lots of reports have been presented to show the enzyme-mimicking properties of carbon nanomaterials [15,16,17,18]. Among all the carbon-based nanomaterials, fullerene and its derivatives were the first nanomaterials examined for their enzyme-like properties [19,20]. C_60_[C(COOH)_2_]_2_ is an example of such a type of fullerene which catalyzes TMB in the presence of H_2_O_2_ [21]. The peroxidase-like activity of graphene and its derivatives has also been largely exploited, indicating its great potential in mimicking peroxidase. Qu et al. were the first to explore the intrinsic peroxidase-mimicking activity of graphene oxide [16]. High surface area values and affinity towards organic substrates make graphene oxide even more efficient than natural HRP towards TMB. Carbon nanotubes are also widely exploited for their enzyme-like properties. Metal catalysts are usually used for the synthesis of single-walled carbon nanotubes (SWNTs) and sometimes traces amounts remain in the product, so the enzymatic activity of SWNTs could be due to these metal residues. To address this concern, these trace amounts of metal were removed from SWNTs by sonication in concentrated sulfuric and nitric acids, and the treated SWNTs still retained their enzyme-like activities, which confirmed that the catalytic activity was due to SWNTs rather than trace amounts of metal catalyst [17]. 

### 3.2. Metal-Based Nanozymes

After the first report of the intrinsic enzyme-like activity of Au nanoparticles in 2004, metal nanomaterials have been extensively studied as potential candidates for enzyme mimics [7,22]. Metal nanomaterials (such as Au, Pd, Pt, Ag, Bi, etc.) with intrinsic enzyme mimicking activities have some special features, such as their multi-enzyme mimicking activities being pH and temperature dependent, the fact their activities could be enhanced by the plasmonic properties of noble metal nanomaterials, and their enzyme-mimicking activities being tuned when they form alloys with other metals, e.g., in bimetallic nanostructures [23,24]. 

Peroxidase-like activity of gold nanoparticles was observed by Chen and co-workers. Through their extensive study and by comparison of the peroxidase-like activity of unmodified, amino-modified, and citrate-capped gold nanoparticles, it was revealed and confirmed that peroxidase-like activity was indeed contributed by the gold content of the nanoparticles [25]. Gold nanoparticles with different surface charges (positive or negative) have been shown to exhibit peroxidase-mimicking activity [26]. The enzyme like activities of gold nanoparticles are microenvironment dependent, and they can be changed or tuned by changing the pH or surface modification resulting in changed affinities between nanozymes and substrate. Li et al. have demonstrated pH-switchable peroxidase and catalase mimic activities of Au, Ag, Pt and Pd nanozymes on the basis of computational studies. Nanozymes exhibited peroxidase-like activities at acidic pH and catalase-like activities at basic pH [26]. Wang et al. reported peroxidase-like activities of bovine serum albumin (BSA)-encapsulated fluorescent gold nanoclusters [27]. Platinum nanoparticles (1–2 nm) were prepared which exhibited dual enzyme mimic behaviors (catalase and peroxidase) in different microenvironments (depending on pH and temperature) with high stability [28]. Peroxidase-mimicking capability of 10 nm Pt nanocubes stabilized by cetyltrimethylammonium bromide (CTAB) was also demonstrated [29]. There are many reports on the enzyme-like properties of bimetallic nanomaterials. Peroxidase-like activity of bismuth—gold nanoparticles was demonstrated by Lien et al. [30]. Bimetal nanoparticles like Au@Pt nanorods were examined by He et al., who demonstrated that Au@Pt nanorods had multiple enzyme-mimetic capabilities [31]. Silver alloys with Au, Pd and Pt also possesses intrinsic peroxidase-mimic properties and can oxidize colorimetric substrates to the corresponding products with H_2_O_2_ [32].

### 3.3. Metal Oxide-Based Nanozymes

Metal oxide-based nanozymes with peroxidase-like activity have been extensively investigated by researchers because of the ease of fabricating colorimetric detection systems which generate the corresponding colorimetric signal in the presence of hydrogen peroxide (H_2_O_2_). Yan et al. reported for the first time the intrinsic peroxidase-like activity of Fe_3_O_4_ MNPs of three different sizes (30, 50 and 300 nm). Nanozymes with smaller sized particles provide more exposed surface area for catalysis, so they exhibit higher activity, a large surface area for surface chemistry, stability at a wide range of temperatures, workability in a wide pH range, robustness, cost effectiveness and large scale production [33]. After this pioneering report Wei and Wang developed novel sensing platforms with Fe_3_O_4_ MNPs as peroxidase mimics [34]. Some doped ferrites like bismuth and europium-doped FeO_3_ and cobalt, manganese and zinc doped Fe_2_O_4_ have also been explored as peroxidase mimics [35,36,37,38,39,40,41,42].

Cerium oxide nanomaterials or nanoceria also have been widely explored for mimicking natural enzymes [7,43,44]. The variable valence states of Ce^3+^ and Ce^4+^ and the mobile lattice oxygen in nanoceria make it highly efficient for catalytic applications [45]. Intrinsic peroxidase-mimicking activities of nanoceria have been disclosed in various reports [44,46,47]. Nanoceria has high efficacy to catalyze peroxidase’s substrates in the presence of H_2_O_2_ [44,48]. Vanadium oxide-based nanomaterials have also got a lot of attention from researchers in recent years. Enzyme-like activities were observed for vanadium oxide nanomaterials and further exploited for biosensing, antibiofouling, and cytoprotection applications [35,36,37,38]. Tremel et al. demonstrated that V_2_O_5_ nanowires possess intrinsic peroxidase like activity and can mimic natural vanadium haloperoxidase [35,36]. Cobalt oxide nanomaterials have been also reported as nanozymes to mimic natural enzymes like peroxidase, catalase, SOD, etc. Co_3_O_4_ is one of the very efficient nanozymes among cobalt oxide- based nanozymes which exhibited higher enzyme-mimicking activities when compared to Fe_3_O_4_ nanoparticles showing enzyme-like properties. In its enzyme-like activity phenomena, Co_3_O_4_ undergoes a Co^2+^→Co^3+^→Co^2+^ regeneration mechanism [39,40].

Enzyme-mimicking activities of copper oxide nanomaterials are also reported for different sensing applications. Chen et al. observed peroxidase-like activity in CuO nanoparticles [49,50]. A comparative study of the enzyme kinetics of a CuO-based nanozyme with natural HRP and other artificial nanozymes (e.g., Fe_3_O_4_- and FeS-based peroxidase mimics) revealed that the CuO nanozyme had higher catalytic activity toward TMB [51]. Hu et al. reported cupric oxide nanoparticles (CuO) as peroxidase mimics [52]. TiO_2_ nanotube arrays mimicking peroxidase activity were fabricated by Dong et al. In the same fashion, ZnO, MnO_2_ nanowires, and NiO NPs, have also been reported for their peroxidase mimicry [53,54,55]. 

### 3.4. Other Nanomaterial for Nanozymes

Some other nanomaterials have also been explored for mimicking natural enzymes beyond carbon-based nanomaterials, metal-based nanomaterials and metal oxide-based nanomaterials. Metal-organic frameworks (MOFs) and MOFs loaded with other catalysts have been reported to exhibit enzyme-like properties [56,57,58,59]. Liu et al. reported a nanosized porous metal–organic framework, Fe-MIL-88NH_2_ exhibiting intrinsic peroxidase-like activity and used it for colorimetric detection of glucose [59]. Qin et al. demonstrated a hemin@metal-organic framework mimicking peroxidase and applied it for glucose detection. Prussian Blue, [Fe(III)Fe(II)(CN)_6_]^−^, has been also explored to mimic peroxidases. In their earlier study Gu et al. showed that a Prussian Blue coating could tune the peroxidase-mimicking activity of γ-Fe_2_O_3_ nanoparticles, but later they also found enzyme-like activities of Prussian Blue nanoparticles [58,59]. They also demonstrated that the enzyme-mimicking properties of Prussian Blue nanoparticles were dependent on the microenvironment. For instance, nanoparticles exhibited peroxidase-mimicking activity at acidic pH and catalase-mimicking activity at a basic pH.

Metal hydroxides have gained much attention in recent years as artificial enzymes. In most of the demonstrations metal hydroxides showed peroxidase-like activities [60,61,62,63]. Peroxidase-like activity of CoFe-layered double hydroxides was reported by Sun et al., who further used CoFe hydroxides for colorimetric detection of H_2_O_2_ and glucose [64]. Recently Tan et al. reported a very efficient peroxidase-mimic system based on nanocages of Cu(OH)_2_, which showed more peroxidase-like activity than natural enzymes [65]. Metal chalcogenides are another class of nanomaterials which has been explored for their enzyme-like activities. Some examples include CuS, MnSe and FeSe which mimic peroxidase. 

## 4. Application of Nanozymes in Colorimetric Sensing of Glucose

Nanozymes can be applied as a single component or multicomponent systems towards colorimetric detection of glucose. Single component systems are based on a single nanozyme material, while multicomponent systems include nanocomposites, doped and functionalized nanomaterials to synergize the oxidase-like activity.

### 4.1. One Component System

With the discovery of ferromagnetic nanoparticles, single component nanomaterials such as noble metal NPs, metal oxides, ceria nanoparticles, and carbon-based nanostructures etc. have been explored for their intrinsic oxidase/peroxidase-like properties [18,23,31,33,50,66,67]. All those single component materials possessing peroxidase-like catalytic activities were utilized for the colorimetric detection of H_2_O_2_ and glucose.

**Figure 2:**
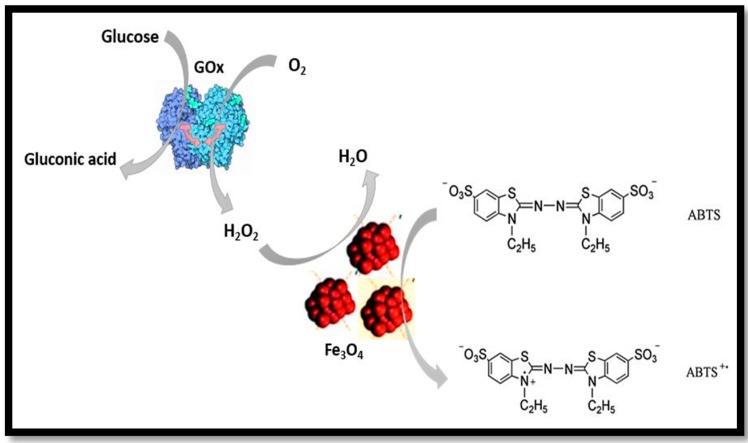
Colorimetric detection of H_2_O_2_ and glucose based on Fe_3_O_4_ nanozyme as peroxidase mimic.

In this context, Wang and co-workers used the novel properties of Fe_3_O_4_ MNPs as a peroxidase mimetic for the colorimetric detection of H_2_O_2_ and glucose [34]. The working principle of this assay is demonstrated in Figure 2. The obtained limit of detection (LOD) for glucose was as low as 30 µM with a linear range of 50–1 × 10^3^ µM. This colorimetric method for glucose detection showed good selectivity over different glucose analogues (e.g., fructose, maltose, and lactose). 

This work led to the exploration of other nanozymes for glucose monitoring. Li and co-workers synthesized positively charged AuNPs. The positively charged AuNPs catalyzed the oxidation of the peroxidase substrate TMB in the presence of H_2_O_2_ to produce a blue color [67]. Additionally, AuNPs were observed to enhance the activities of glucose oxidase (GOD) and horseradish peroxidase [68,69]. The mechanism of catalytic activity of AuNPs was based on the fact that H_2_O_2_ was absorbed on the surface of AuNPs and the O-O bond in H_2_O_2_ were broken up into double ^•^OH radicals. By a partial electron exchange interaction, the generated ^•^OH radicals were stabilized by AuNPs. This mechanism contributed to the catalytic ability of AuNPs. The LOD of this proposed assay for glucose was 4 µM, which was lower than that obtained using Fe_3_O_4_ magnetic nanoparticles as peroxidase mimetic [34]. 

**Figure 3:**
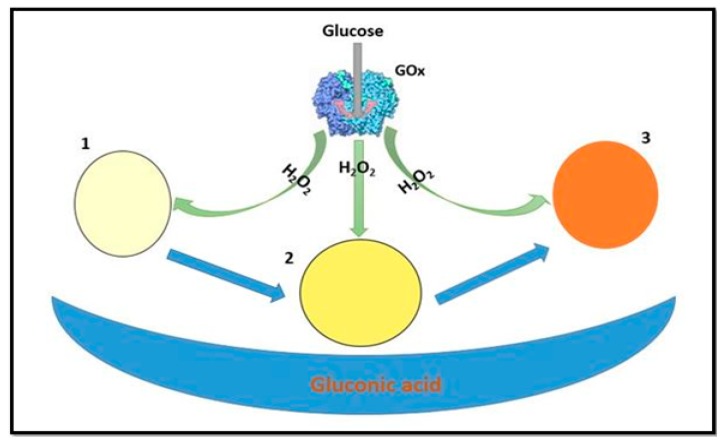
Ceria Paper based, colorimetric glucose sensor, color change of nanoceria-based filter paper from white (1)-yellow (2) to dark orange (3) [70].

Considering the cost effectiveness of paper-based platforms, glucose detection was also performed on a common and cheap Whatman filter paper (no 1) by integrating nanozymes. In this regard, Andreescu et al. used nanoceria as a colorimetric probe in a bioanalysis for the detection of H_2_O_2_ and glucose [70]. Glucose oxidase (GOx)-biofunctionalized ceria paper was employed for quantitative detection of glucose. The working principle of the ceria bioassay was based on the production of H_2_O_2_ by glucose oxidase in the presence of glucose, followed by a change in the surface chemistry of the nanoceria nanoparticles due to H_2_O_2_ causing conversion of Ce^3+^ to Ce^4+^ [71], accompanied by a color change from white-yellow to dark orange. As a demonstration, the working principle of the assay is shown in Figure 3. The analytical performance of the assay was dependent on the nanoceria concentration. The designed sensor was based on the co-immobilization of the nanoceria and GOx onto the filter paper. The achieved LOD for glucose sensing was 500 µM, with a linear range from 5 × 10^2^–1 × 10^5^ µM. This method was also applied in serum samples to determine the glucose concentration. Lv and co-workers synthesized some well-redispersed ceria nanoparticles [72]. The synthesized CeO_2_ NPs were characterized by good dispersion properties and excellent peroxidase-like activity. However, ceria nanoparticles have wide size distributions, a tendency to agglomerate, and poor dispersivity in aqueous media, which may limit their practical applications [73,74]. The synthesized ceria nanoparticles have also been used for the detection of glucose in aqueous medium [72]. The designed assay permitted a LOD of 3 µM, with a linear glucose detection range from 6.6–130 µM. This sensitive and highly selective colorimetric assay was applied for glucose determination in human serum. The achieved analytical figures of merits in term of LOD and linear range were better than those obtained with the paper-based platform, however, the nanoceria paper-based assay offers the advantages of portability, stability and suitability for onsite analysis.

Graphitic structural materials like carbons dots (CDs) have been widely investigated in the last two decades [75,76]. C-dots have biocompatibility, small size, and low toxicity, and remain stable for more than one year at 4 °C [77,78,79]. CDs possess the ability to behave as either excellent electron acceptors or electron donors. C-dot quantum confined fluorescent carbon materials have been widely employed as biosensor agents [80]. Subsequently, the intrinsic peroxidase-like activity of C-dots was used for the colorimetric detection of H_2_O_2_ and glucose [81]. A LOD of 0.4 µM with a linear range from 1–5 × 10^2^ µM was achieved with this method. Based on the designed working principle of this colorimetric assay, glucose was detected in serum samples. 

**Figure 4:**
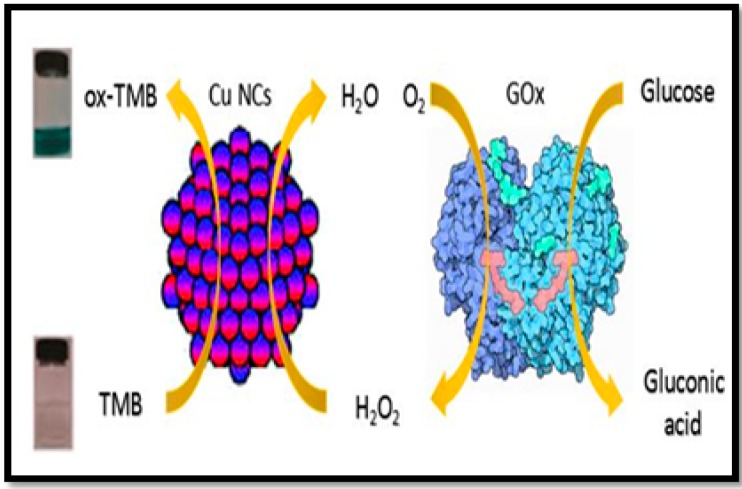
Colorimetric detection of glucose by using glucose oxidase (GOx) and a Cu NCs-catalyzed color reaction [91].

Cupric oxide nanoparticles are more stable and possess unchanged catalytic activity over a wide range of pH and temperature values, although the aggregation and settlement of the commercial CuO NPs in aqueous media will affect certain applications [82]. The colorimetric detection of glucose was performed in a one-step method based on the enzyme-like properties of water soluble cupric oxide [49]. The linear range for glucose detection was from 1 × 10^2^–8 × 10^3^ µM. Metal nanoclusters possessing low toxicity and ultrafine size have been used as a promising candidate with fascinating catalytic characteristics in the field of molecular imaging, biosensors, and catalysis [83,84,85,86,87,88,89,90]. In this context, copper nanoclusters (Cu NCs) were used as a one-component nanomaterial for the colorimetric detection of glucose [91]. The working principle of the method is shown in Figure 4. This assay was characterized with a LOD of 100 µM, while presenting a linear range of 1 × 10^2^–2 × 10^3^ µM.

Xia and their co-workers investigated a homogeneous system of silver nanoprisms with GOx for the simple, sensitive and low-cost colorimetric sensing of glucose to control diabetes mellitus [92]. The silver nanoprisms possess highly reactive edges/tips, strong tip sharpness and aspect ratio-dependent surface plasmon resonance absorption which enhances the detection limit. 

This method enabled the visual detection of glucose (using a blue to mauve color change) with the naked eye in the range from 0.2–1 × 10^2^ µM with a LOD of 0.2 µM, lower than that obtained with hybrid (metal-NP)-GOx systems [93,94,95]. The sensitivity of the system could be attributed to the highly reactive edges/tips and strong tip sharpness and aspect ratio of the Ag nanoprisms used. This proposed sensing platform was also applied in serum samples to detect glucose concentration. Liu et al. designed a sensing platform for glucose based on the GOx-catalysed growth of small sized AuNPs in the presence of glucose [96]. The size of AuNPs played an important role in the colorimetric detection of glucose. The LOD was 49 µM with a linear range from 1×10^2^–1×10^3^ µM of this method. This colorimetric assay was successfully applied to measure glucose in serum glucose. An analytical performance comparison of the single component nanozyme methods for the detection of glucose is summarized in Table 1.

### 4.2. Multi- Component System

In the area of nanomaterials, much progress has been accomplished due to incredible achievements in nano-research and the intrinsic characteristics of nanomaterials [9,97,98,99,100,101]. In the last decade, the trend is shifting towards the multi-component nanozymes because most of the single component artificial nanozyme enzyme mimetics are characterized with low catalytic activity, poor dispersion and precipitation under typical complex physiological conditions [102,103,104]. Furthermore, the catalytic properties of these nanozymes are highly dependent on the size, shape and geometry of the nanostructures [7]. Researchers have therefore endeavored to design multi component systems by integrating multiple functionalities into a single nanozyme system [105]. 

Carboxyl functionalization is reported to synergize the oxidase-like properties of nanozymes in the construction of colorimetric assays. For example, the carboxyl-modified graphene oxide (GO-COOH) has been shown to be a peroxidase mimetic for the colorimetric detection of glucose [16]. The low-cost, good stability, resistance to denaturation, high surface-to-volume ratios as well as the high affinity for organic substrates through π-π and hydrophobic interactions of GO-COOH makes them a superior candidate as compared to natural HRP and Fe_3_O_4_ nanozyme. The achieved LOD for glucose was 1 µM, with a linear range from 1–20 µM. The designed method was applied to determine glucose in blood serum. In parallel, Shu and co-workers synthesized C_60_-carboxyfullerene C_60_[C(COOH)_2_]_2_ and designed a sensitive and selective colorimetric assay for glucose detection by exploring the oxidase-like properties of this novel functionalized material [15]. The facile modification of fullerene-C_60_ with carboxyl groups improved its solubility in water [106]. The obtained LOD with this assay was 0.5 µM. The practicability of this assay was explored by the detection of glucose in human serum. In the same context, Wang and co-workers reported that a silver nanoparticles on graphene quantum dots (GQDs/AgNPs) hybrid exhibits a superior absorbance fading response for the reduction of H_2_O_2_ [107]. The GQDs acts as an excellent stabilizer in the GQDs/AgNPs hybrid, with a nanohybrid stability period of one week. Sensitive and selective colorimetric detection of glucose was performed based on the color fading of the GQDs/AgNPs hybrid in combination with the generated H_2_O_2_. The LOD of this assay was 0.17 µM, while the linear range was from 0.5–400 µM. In another study, chitosan-stabilized nanoparticles (Ch Ag NPs) were successfully synthesized by Huang and co-workers and used for the colorimetric detection of H_2_O_2_ and glucose [66]. The Ch-Ag NPs have high surface area, stability and the matrix material prevents the aggregation of the nanoparticles. The linear range was from 5–200 µM and the obtained LOD was 0.1 µM. The obtained LOD was lower than that obtained with various other nanoparticles used as peroxidase mimetics. Using this method, the glucose level was detected in blood serum. Similarly, Tseng et al. synthesized poly(diallyldimethylammonium chloride)-coated Fe_3_O_4_ NPs and found that PDDA-Fe_3_O_4_ not only has peroxidase-like activity but also has the ability to adsorb GOx through electrostatic attraction [108]. The synthesized GOx-Fe_3_O_4_ composite was used for seven repeated cycles with a 1.1-fold decrease in absorbance output signal in the optical detection of glucose. The LOD was 30 µM, while a linear range from 30–1 × 10^3^ µM was achieved with this method. 

In a subsequent study, Chen and co-workers explored the intrinsic peroxidase-like activity of ZnFe_2_O_4_ magnetic nanoparticles (MNPs) [41]. ZnFe_2_O_4_ MNPs exhibited good catalytic properties, stability, dispersibility, and rapid separation compared to other peroxidase nanomimetics and HRP. The linear range was from 1.25–18.75 µM, and the obtained LOD was 0.3 µM with this assay. This colorimetric assay was also applied to detect glucose in urine sample of patients with diabetes. In the same context, Adhikary et al. synthesized a Prussian Blue-modified iron oxide (PB-Fe_2_O_3_) nano-composite and utilized it for the colorimetric detection of glucose [109]. The peroxidase-like activity of Fe_2_O_3_ has been enhanced by impregnating Fe_2_O_3_ with Prussian Blue, which shows high catalytic activity towards peroxidase substrates. The achieved LOD of this assay was 0.16 µM, with a linear range from 1 to 80 µM. The glucose concentration was determined in blood serum applying this assay. In addition, Kemin and co-workers synthesized a new type of magnetic mesoporous silica nanoparticles (Fe_3_O_4_@MSN) with Fe_3_O_4_ as the core and a mesoporous silica shell [110]. The synthesized magnetic mesoporous silica nanoparticles were shown to exhibit peroxidase mimic activity. A LOD of 4 µM with a linear range from 10–500 µM was achieved with this assay.

Zhang and their co-workers have synthesized CF nano-cubes having hierarchical nanostructures [111]. Hierarchical materials possess a high surface to bulk ratio, and also provide more active sites useful for catalysis. The obtained LOD with this assay was 2.47 µM, with a linear range from 8 to 90 µM. This colorimetric method was applied in serum samples to detect glucose. In the same manner, Guo’s group revealed that apoferritin paired gold clusters (Au-Ft) possess intrinsic peroxidase-like catalytic activity [112]. The apoferritin paired gold clusters (Au-Ft) can provide an enzyme active center, thereby facilitating the ability of substrate molecular binding and also stabilize the enzyme-substrate complex. The linear range was from 2 × 10^3^–1 × 10^4^ µM. Xu and co-workers demonstrated the optical detection of glucose through a homogenous system containing DNA-embedded core-shell Au@Ag NPs [113]. This assay permitted a LOD of 0.01 µM, with a linear range from 0–2 × 10^2^ µM. Glucose was determined in fetal bovine serum by utilizing this optical biosensor. Ai and co-workers synthesized FeSe-Pt@SiO_2_ nanospheres and explored the peroxidase-like catalytic activity [114]. The achieved LOD for glucose sensing was 1.136 nM. Similarly, Ying and his co-workers synthesized a symmetric hematite-silica hybrid of Janus γ-Fe_2_O_3_/SiO_2_ nanoparticles (JFSNs) and used it for the colorimetric detection of H_2_O_2_ and glucose [115]. JFSNs exhibit intrinsic peroxidase-like activity, which is a higher and more stable over a wide range of pH and temperature values compared with the natural enzyme HRP. Furthermore, the JFSNs offer a multiple functions platform for biosensing, due to their unique asymmetric structure. The LOD for this proposed assay was 3.2 µM. This method was also used for the determination of glucose in serum samples. Liu et al synthesized a V_2_O_3_-ordered mesoporous carbon composite (V_2_O_3_-OMC). A facile analytical method was developed to detect glucose by using V_2_O_3_-OMC and glucose oxidase [116]. The linear range was from 10–4 × 10^3^ µM and the LOD for glucose sensing was 3.3 µM. This developed assay showed good sensitivity and high selectivity and enough reliability in real samples. 

**Figure 5:**
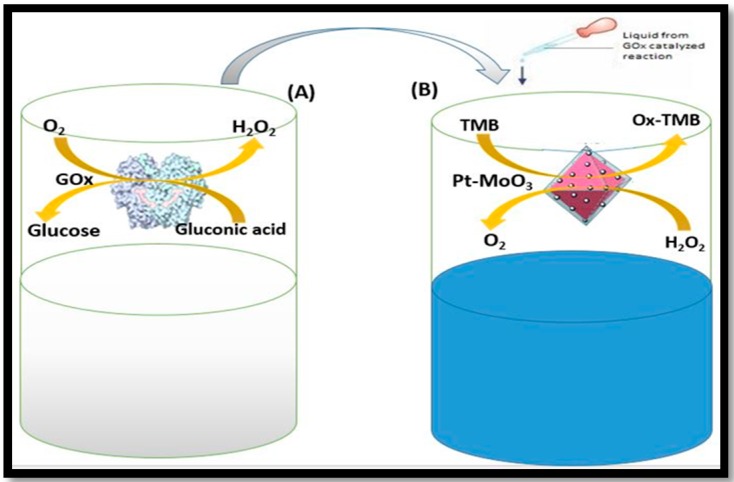
Colorimetric detection of glucose by using GOx catalyzed reaction (**A**) and Pt-MoO_3_ hybrid nanomaterials as catalysts (**B**) [118].

Liu and co-workers synthesized NiO NPs modified with 5,10,15,20-tetrakis (4-carboxyphenyl)-porphyrin (H_2_TCPP) [H_2_TCPP-NiO nanocomposites] [53]. The obtained LOD of proposed assay was 20 µM, with a linear range from 50–5 × 10^2^ µM.Doped nanozymes are also considered a class of multi-component system. For example, Chen et al. synthesized nitrogen-doped graphene quantum dots (N-GQDs) and explored how the produced N-GQDs has high intrinsic peroxidase-like catalytic activity [117]. The N-GQDs have a large surface area ratio and more active sites along with the additional characteristics of low cost, excellent dispersibility in water, stability against harsh conditions, and tunable catalytic activities. The LOD was 16 µM and a linear range from 25–375 µM was achieved with this assay. This assay was successfully applied in the detection of glucose in blood serum. Subsequently, Ying and co-workers synthesized platinum NPs with sizes from 1–3 nm and uniformly grew them on a molybdenum trioxide (MoO_3_) nanosheet surface. It was observed that Pt-MoO_3_ have a peroxidase mimic activity [118]. The working principle of the assay performed is shown in Figure 5. The LOD of this assay was 0.1874 µM, while the linear range was from 5–500 µM. The colorimetric assay was successfully applied to determine the glucose concentration in serum samples. Table 2 summaries the analytical characteristics of the multicomponent nanozyme systems for the detection of glucose.

## 5. Conclusions and Perspectives

Nano-receptor-based methodologies offer a novel and attractive paradigm in terms of new and augmented functionality for the optical detection of glucose. These nano-receptors are characterized by various advantages which include, but are not limited to, low cost, facile preparation, large scale synthesis, high stability and sustained catalytic activities. Optical glucose biosensors based on nanozymes are characterized by high sensitivity that may be attributed to the large surface area per volume of the nanomaterials. Natural oxidase/peroxidase enzymes are proteinic in nature and their analytical figures of merits are highly dependent on the characteristics of the medium such as pH and temperature, while nanozyme-based sensors are independent of such characteristics. The selectivity of the nanozyme-based optical sensors can be considered less as compared to that of natural enzymes. The highly reactive surface of the nanomaterial such as ceria nanoparticles and nonspecific adsorption of interfering molecules on the particle surface such as that of gold/silver nanoparticles may result in false positive or negative results. However, these selectivity issues are limited, as glucose oxide enzyme is very selective enzyme towards conversion of glucose into hydrogen peroxide.

This review provided a brief survey of the different types of nanomaterials which are employed as potential receptor elements to replace natural enzymes in the field of biosensors, and have found widespread applications for optical detection of glucose. These nanosystems were initially explored as single component systems, and were successfully employed to design colorimetric sensors. Generally, most of the single component artificial nanozyme mimetics are characterized by low catalytic activity, poor dispersion and precipitation under complex physiological conditions [102,103,104]. 

Furthermore, the catalytic properties of these nanozymes are highly dependent on the size, shape and geometry of the nanostructures [7]. With further advancements in the field of nanotechnology, researchers have therefore endeavored to design multi-component systems by integrating multiple functionalities into a single nanozyme system [105]. However, controllable synthesis of the multi-component systems presents tremendous challenges and problems in their enzyme-mimicking colorimetric sensing applications. The major limitations associated with multi- component systems include, but not limited to, restrictions in the partial properties of each component by interface interactions, decreased catalytic properties of the core component and unfavorable structural and chemical arrangement of functional components. It is widely accepted that the preparation of multi-component nanosystems requires tedious and time consuming synthesis process involving highly toxic solvents. 

Moreover, nano-receptor materials do not have real enzyme-like properties and it is not possible to regenerate the nanomaterial surface in most of the cases for subsequent measurement, limiting their applications in amperometric biosensors or for repeated assays. Some attempts were made to regenerate the nano-receptor surface for repeated cycles, but a decrease in the catalytic efficiency was observed after eight cycles [108]. Moreover, controlling the reactivity of nanomaterials against certain interfering molecules is a very difficult task which may result in the generation of nonspecific signals, thus affecting the assay selectivity and specificity. The reactivity of nanomaterials is mainly related to the functional groups of the analytes, and closely related interfering molecules share a very similar structure to the analyte of interest and have possibility to react with the nanomaterials. This reactivity may result in generation of signals even in the absence of analyte and produce false positive results. 

To replace enzymes for biosensing applications, it is highly desirable to design selective and specific nanomaterials to overcome the matrix interferences. Moreover, future research may focus on the methods to regenerate the nano-surface to increase the reusability of the nano-sensors. 

## Figures and Tables

**Table 1:** Analytical performance comparison of the single component nanozyme methods for the detection of glucose.

Nanozymes	Limit of Detection (LOD)	Linear Range	Real Sample Test	Ref
Fe_3_O_4_ MNPs	30 µM	50–1 × 10^3^ µM	N/A	[34]
Positively-charged AuNPs	4 µM	18–1100 µM	N/A	[67]
Nanoceria	500 µM	5 × 10^2^–1 × 10^5^ µM	Human Serum	[70]
C-dots	0.4 µM	1–5 × 10^2^ µM	Human Serum	[81]
Water soluble CuO NPs	N/A	1 × 10^2^–8 × 10^3^ µM	N/A	[49]
Re-dispersed CeO_2_ NPs	3 µM	6.6–130 µM	Human Serum	[72]
Copper nanoclusters	100 µM	1 × 10^2^–2 × 10^3^ µM	N/A	[91]
Ag nanoplates	0.2 µM	0.2–1 × 10^2^ µM	Human Serum	[92]
AuNPs	49 µM	1 × 10^2^–1 × 10^3^ µM	Human Serum	[96]
MPs	3.74 µM	N/A	N/A	[32]

**Table 2:** Analytical performance comparison of the multi component nanozyme methods for the detection of glucose.

Nanozymes	Limit of Detection (LOD)	Linear Range	Real Sample Test	Ref
GO-COOH	1 µM	1–20 µM	Human Serum, juices	[16]
Ch-Ag NPs	0.1 µM	5–200 µM	Human Serum	[66]
PDDA-Fe_2_O_3_	30 µM	30–1 × 10^3^ µM	Human Serum	[108]
ZnFe_2_O_4_ MNPs	0.3 µM	1.25–18.75 µM	Urine sample	[41]
C_60_[C(COOH)_2_]_2_	0.5 µM	N/A	Human Serum	[21]
PB-Fe_2_O_3_	0.16 µM	1–80 µM	Human Serum	[109]
Fe_3_O_4_@MSN	4 µM	10–500 µM	N/A	[110]
GQDs/AgNPs	0.17 µM	0.5–400 µM	N/A	[107]
CF nano-cubes	2.47 µM	8–90 µM	Human Serum	[111]
Apoferritin paired gold clusters (Au-Ft)	N/A	2 × 10^3^–1 × 10^4^ µM	N/A	[112]
DNA-embedded core-shell Au@Ag NPs	0.01 µM	0–2 × 10^2^ µM	Fetal bovine serum	[113]
FeSe-Pt@SiO_2_ nanospheres	1.136 nM	0.01136–227 µM	Human Serum	[114]
V_2_O_3_-OMC	3.3 µM	10–4 × 10^3^ µM	Serum	[116]
Janus γ-Fe_2_O_3_/SiO_2_ NPs	3.2 µM	0–20 µM	Human Serum	[115]
H_2_TCPP-NiO nanocomposites	20 µM	50–5 × 10^2^ µM	N/A	[53]
Nitrogen-doped graphene quantum dots	16 µM	25–375 µM	Serum	[117]
Pt-MoO_3_ hybrid nanomaterials	0.1874 µM	5–500 µM	Serum	[118]
